# The mental health of parent versus non-parent post-secondary students

**DOI:** 10.1371/journal.pmen.0000021

**Published:** 2024-08-12

**Authors:** Katie J. Shillington, Julia Yates, Tara Mantler, Jennifer D. Irwin

**Affiliations:** 1 Faculty of Health Sciences, Health and Rehabilitation Sciences, The University of Western Ontario, London, Ontario, Canada; 2 Department of Neurobiology, University of California San Diego, San Diego, California, United States of America; 3 Center for Empathy and Social Justice in Human Health, T. Denny Sanford Institute for Empathy and Compassion, University of California San Diego, San Diego, California, United States of America; 4 Faculty of Science, Department of Psychology, Wilfrid Laurier University, Waterloo, Ontario, Canada; 5 Faculty of Health Sciences, School of Health Studies, The University of Western Ontario, London, Ontario, Canada; Xi'an Jiaotong-Liverpool University, CHINA

## Abstract

Post-secondary students experience abnormally high levels of stress compared to the general population, and parents pursuing post-secondary education have additional demands that challenge their mental health. Understanding the mental health of parent versus non-parent students is imperative to support students in their academic pursuits. The purpose of this mixed-methods, cross-sectional paper was two-fold: (1) to investigate the mental health (flourishing/languishing) of parents pursuing post-secondary education in Ontario, Canada with children/youth aged 0–18 years compared to non-parent post-secondary students; and (2) to explore parents’ and non-parents’ lived experiences of mental health (flourishing/languishing) while pursuing post-secondary education. A total of 374 students (*n* = 86 parents; *n* = 288 non-parents) completed an online survey that included demographics and the previously validated Mental Health Continuum-Short Form. A subset of participants (*n* = 10 parents; *n* = 10 non-parents) participated in one of six focus groups (3 parent focus groups; 3 non-parent focus groups). Results from the independent sample t-tests indicated no significant differences between the overall mental health scores. Thematic analysis revealed 4 themes for student-parents: (1) factors that challenge mental health; (2) realities of being a student-parent; (3) social connectivity among family and friends; and (4) mental health fluctuations. A total of 6 non-parent specific themes were found: (1) social connectivity among peers; (2) factors that challenge mental health; (3) the bidirectional relationship between school and mental health status; (4) prioritizing academic roles; (5) finding purpose through academic pursuits; and (6) admiration for student-parents. Findings from the current study highlight the dichotomy in student-parent versus non-parent academic identities and are important for university personnel to understand to provide tailored supports.

## Introduction

Post-secondary students experience unique stressors that transcend academic, social, and financial domains [1]. Notably, students have described relationship challenges, financial struggles, difficulty balancing school and life commitments, concern regarding future career prospects, and pressure to succeed, all of which contribute to poor mental health [2]. These stressors are particularly worrisome because the majority of post-secondary students fall into the age group most at-risk for the development of mental illness [3,4]. It is not surprising then, that the prevalence of psychological distress, mental illness diagnoses, and help-seeking for mental health related challenges has increased in recent years (2013–2019) among post-secondary students [1]. Specifically, Linden and colleagues [1] used data from the 2013, 2016, and 2019 National College Health Assessment (NCHA) surveys to examine mental health trends among Canadian post-secondary students. The authors concluded that students’ stress levels increased, and anxiety and depression were the most reported mental disorders they experienced [1]. Additionally, the American College Health Association [5] surveyed 78,024 Canadian students and found that in Spring 2023, 71% of students reported moderate to serious psychological distress and 40% of students reported that stress impeded their academic performance. These findings are consistent with Duffy and colleagues [6], who surveyed over 1500 first-year university students in Canada and concluded that the proportion of students who screened positive for anxiety and depressive symptoms increased over the course of their first year, from 33% to 39% and 28% to 36%, respectively. Similarly, Fried and colleagues [7] assessed the mental health of full-time undergraduate and graduate students (*N* = 792) at a Canadian university and concluded that the anxiety levels of participants in their study were concerning. Further, Wiens and colleagues [8] used data from the Canadian Community Health Surveys (2011–2017) to assess the mental health status of post-secondary students aged 18–25. The authors concluded that the prevalence of experiencing low mental health and mood and anxiety disorders increased among female post-secondary students over time. Additionally, Linden and colleagues [9] assessed the stress of Canadian post-secondary students (*N* = >10,000) over the course of an academic year (2020–2021) and concluded that stress and psychological distress were high at the beginning of the year and remained high throughout the year. While it is evident that post-secondary students experience stressors that challenge their mental health in general [1,2,6], parents pursuing post-secondary education face additional demands while navigating their multiple roles [10–12].

Little research exists on parents pursuing post-secondary education (hereafter referred to as student-parents) in Canada. Though dated, van Rhijn and colleagues [13] provide the most recent data on the topic. The authors investigated the enrollment patterns and demographic characteristics of Canadian student-parents [13] and found that enrollment of student-parents increased between 1975 to 2005 (from 11% to 16%, respectively) [13]. Additionally, from 2005 to 2021, the total number of students enrolled in post-secondary education in Canada increased by approximately 19% [14], and in 2014, more than one in four undergraduate students in the United States were raising children [15]. While no research has been conducted on the enrollment status of Canadian student-parents since 2005, it is hypothesized that the number of parents pursuing post-secondary education has increased over the years, based on trends previously described [13]. Thus, if current trends mirror those in earlier years, it is reasonable to believe that the proportion of student-parents in Canadian post-secondary institutions has also increased since 2005. Understanding student-parents is particularly important, given their unique needs, as discussed below [11–12].

Often referred to as “invisible students” [10,11,16,17], the needs of parents pursuing post-secondary education consistently go undocumented [12,18]. Namely, Moreau [17] emphasized the ways in which academia has excluded certain groups. The author examined written policies and documentation across 10 university case studies and found that student-parents and children were subject to feelings of invisibility and ‘othering’ in academic settings [17]. Specifically, Moreau [17] concluded that institutions had varying policies regarding whether children were allowed on campus with their parent(s). As a result, student-parents have been made to feel invisible in institutional policies and physical environments [16]. Similarly, Sallee and Cox [12] illuminated the “invisible student” phenomenon in their qualitative work, wherein student-parents (*n* = 27) in the United States described difficulties accessing parent-specific campus-based resources, such as childcare. Participants in this study also described structural barriers to being a student-parent, including a lack of dedicated spaces for breast pumping and policies banning children from specific campus environments (e.g., study spaces) [12]. Lack of childcare among student-parents was also highlighted by Manze and colleagues [19], wherein 30% of student-parents (*n* = 225) in their study sample reported that a lack of childcare interfered with school. These students were also significantly more likely to need mental health treatment compared to those that reported childcare had little to no interference with school [19]. Similarly, in a qualitative study conducted by Kensinger and Minnick [11] student-mothers (*n* = 27) in the United States described a lack of childcare services and financial support. Participants also described stigma associated with being a mother pursuing post-secondary education, such that they felt discriminated against and judged while pregnant [11]. Financial difficulties, structural barriers, and stigma are only some of the stressors specific to student-parents; other stressors include time management challenges [20], difficulties balancing school, work, and parenting [21], and class and childcare schedule conflicts [10,21], all of which can contribute to poor mental health. Additionally, in a study reported in grey literature, Ruff [22] qualitatively explored undergraduate student-parents’ (*N* = 11) experiences of belonging on campus. Participants described a need for connection with other student-parents; while some were able to find social connectivity through shared experiences (e.g., with others in student-parent housing), other participants described feelings of loneliness, which exacerbated the stress already associated with being a student-parent [22]. Participants in this study also spoke to obstacles unique to being a student-parent such as lack of childcare, parenting responsibilities outside of school, and lack of time [22]. Specifically, participants described difficulty balancing parenthood, working, and academic responsibilities, and some described how they would be able to accomplish more if they had consistent childcare [22]. Further, participants underscored the importance of campus resources and accommodations, including being offered parent-specific scholarships and medical leave for having a baby [22]. Taken together, the aforementioned experiences of discrimination and othering, coupled with the structural and systemic barriers that persist in post-secondary institutions, can lead to mental health challenges among student-parents [16].

To date, no research exists on the mental health of student-parents in Canada. Much of the research conducted with student-parents has focused on mothers pursuing post-secondary education and their specific stressors [11,18,23]. Thus, understanding the mental health of student-parents is critical to provide necessary and appropriate supports. To this end, the purpose of this mixed-methods, cross-sectional paper is two-fold: (1) to investigate the mental health (flourishing/languishing) of parents (with children/youth aged 0–18 years) compared to non-parents pursuing post-secondary education in Ontario, Canada; and (2) to explore parents’ and non-parents’ lived experiences of mental health (flourishing/languishing) while pursuing post-secondary education. It is hypothesized that student-parents will report lower mental health scores compared to non-parents, and that a greater proportion of student-parents will have languishing mental health than non-parents. For the purpose of this paper, flourishing mental health is characterized by high levels of emotional, psychological, and social wellbeing, positive emotions, and positive functioning, while languishing mental health represents the opposite side of the spectrum and is characterized by low levels of wellbeing [24,25].

## Methods

### Ethics statement

Ethical approval for the study was received from the host institution’s Non-Medical Research Ethics Board (NMREB #121591). Participants were asked to provide formal consent via an online survey by clicking “Yes, I consent to begin the study.”

### Study design and procedures

The current paper is a part of a larger mixed-methods, cross sectional study titled *The Resilience*, *Health-Related Quality of Life*, *and Mental Health of Parents Versus Non-Parents Pursuing Post-Secondary Education (THRIVE)*. The current paper reports on the mental health data quantitatively measured via surveys and qualitatively explored via focus groups. A subsequent paper including the resilience and health-related quality of life data has been written and submitted elsewhere [26]. Together, these papers provide a broader understanding of the resilience, health-related quality of life, and mental health of student-parents compared to non-parent students in Ontario, Canada.

Participants were recruited from November 2022-February 2023 via social media platforms (i.e., Facebook, Instagram, Twitter, LinkedIn) and mass emails at the host institution. Specifically, the research team contacted social media accounts affiliated with 23 different universities (e.g., Algoma University, Brock University, Queens University, Wilfrid Laurier University, Lakehead University) and 25 colleges (e.g., Algonquin College, Fanshawe College, Humber College, Niagara College, St. Clair College). These accounts were student-oriented and varied in focus including student housing, textbook exchange, residence life, and clubs/societies. Additionally, local parent, mom, and dad social media groups were contacted and asked to share the study advertisement. A total of 987 social media groups were contacted and asked to share the study advertisement. When contacting groups, members of the research team used the same verbiage and study advertisements to maintain consistency. Each group was contacted only one time, as to not overwhelm group administrators. To be eligible for the study, individuals needed to be pursuing a post-secondary degree/diploma at an Ontario institution and be able to read and write in English, and those in the parent group needed to have a child or youth aged 0–18 who lived with them for any period of time. Interested participants were asked to click the link in the study advertisement and/or scan the QR code, which brought them to a Qualtrics^XM^ survey [27] that included the letter of information, eligibility and consent process, as well as the questionnaires. A more fulsome account of the quantitative methods is detailed elsewhere [26].

At the end of the survey, participants had the option to enter a draw for one of three $60 grocery e-gift-cards and were also invited to participate in a focus group. Those who expressed interest in participating in a focus group were provided with the letter of information and asked to submit their availability for a focus group date/time. To reach data sufficiency [28], six focus groups were conducted from March 13–23, 2023 (3 with parents; 3 with non-parents). The focus groups took place via Zoom with a moderator (KS; JY) and an assistant moderator (MS; JC; IT; DV; ZR) and ranged from 60–90 minutes in length. The moderator facilitated the focus group, inclusive of posing the questions, guiding the conversation, and summarizing participant responses. The assistant moderator served as a note-taker and was available to help troubleshoot technological issues, should they arise. Participants were asked to provide verbal consent at the beginning of each focus group and all were audio-recorded. To diminish social desirability bias, at the beginning of each focus group individuals were reminded that there were no right or wrong answers and they could refuse to answer any questions [29]. Data sufficiency was reached by the third focus group for both parents and non-parents. This was achieved through an iterative process wherein the moderators met after each of their respective focus groups to discuss what came out of their focus group conversations, as well as if they were hearing common and/or diverse responses across all focus group participants. Once the moderators concluded they were hearing similar responses from participants and no new perspectives were emerging, data sufficiency was deemed reached. After all focus groups were complete, participants who attended were entered into a draw for one of three $60 grocery e-gift-cards.

### Tools

#### Quantitative

*Demographic questionnaire*. Demographic questions pertained to participants’ age, sex, gender, ethnicity, post-secondary institution, program, faculty, enrolment status, marital status, income, and employment status. Parent-specific demographic questions included the number, age, and living situations of dependent children.

*Mental Health Continuum*. The short form of the Mental Health Continuum (MHC-SF) consists of three items measuring emotional wellbeing, five items to measure social wellbeing, and six items to measure psychological wellbeing [30]. This scale has been previously validated among Canadian adults (Cronbach’s α = 0.82 for emotional and psychological wellbeing; Cronbach’s α = 0.77 for social wellbeing) [31]. Participants were asked to respond to a series of statements on a 6-point Likert scale ranging from 0 to 5 (i.e., never to everyday), with response options reflecting the frequency with which respondents experience symptoms of positive mental health [30]. The MHC-SF also provides a flourishing and languishing mental health indicator based on its subscales. Examples of items include “During the past month, how often do you feel satisfied with life?” and “During the past month, how often do you feel that you had something important to contribute to society?”.

#### Qualitative

*Focus group guide*. The moderators followed a semi-structured focus group guide that was designed for the study and pertained to the mental health of participants. Two focus group guides were developed and utilized: one specific to student-parents (S1 Appendix) and a second specific to non-parent students (S2 Appendix). Notably, as part of the guide, the moderator shared preliminary findings from the MHC-SF (specific to each group) and asked participants the extent to which the findings accurately reflected their mental health, and to offer any insights they wished to share about the findings. The decision to present participants with their group-specific MHC-SF data was purposeful, as the research team was interested in whether the average scores resonated with participants. The research team also wanted to provide participants with a platform and safe space to voice their opinions and provide additional context to study findings. While providing individual participant data might have been more meaningful, this was not possible in a group setting as it would be a breach of confidentiality. In addition to sharing their thoughts on preliminary findings, participants were asked the extent to which their mental health impacted their role as a student (and vice versa), and parents were asked how their role as student-parent impacted their mental health, if at all.

### Data analysis

#### Quantitative

Quantitative analyses were completed via RStudio (Version 12.0; 2020) [32]. Participant demographics were calculated via measures of central tendency and dispersion. Any data missingness was handled using pairwise deletion whereby information was eliminated when the datapoint required for a specific analysis was missing [33].

*Mental Health Continuum*. To calculate a total score on the MHC-SF, item scores were summed (ranging from 0 to 70) [30]. Subscale scores ranged from 0 to 15, 25, and 30 for emotional, social, and psychological wellbeing, respectively. Participants were categorized with flourishing mental health if they reported experiencing ≥ 1 of 3 emotional signs and ≥ 6 of 11 social and psychological signs combined “every day” or “5–6 times a week” [30]. Participants were categorized with languishing mental health if they responded “once or twice” or “never” to 1 or more of the emotional wellbeing questions, and to 6 or more of the 11 social and psychological wellbeing questions [34]. Participants who were neither flourishing nor languishing were categorized as moderately mentally healthy [34]. Higher scores indicated greater levels of positive wellbeing [30].

*Difference between the mental health of parents vs*. *non-parents*. A series of independent sample *t*-tests were run to investigate the difference in (1) overall mental health; and (2) emotional, social, and psychological wellbeing. A two sample Z test of proportions was calculated to assess the differences in the proportion of those classified as flourishing and languishing between parents and non-parents, respectively.

### Qualitative data analysis

To ensure study rigor, strategies to support data trustworthiness (i.e., to support credibility, confirmability, dependability, and potential transferability per Guba & Lincoln [35]) were followed. Specifically, to support data credibility, the moderator reflected back their understanding of what participants said and summarized participant responses to ensure accuracy from the participant perspective. Following the completion of all focus groups, focus group recordings were transcribed verbatim by four research assistants (IT; MS; DV; ZR) and checked for accuracy by a senior research assistant (JC). Focus group transcripts were then organized in Quirkos, a qualitative analysis software [36]. Data was analyzed following the method for thematic analysis outlined by Braun and Clarke [37]. First, two researchers (KS; JY) familiarized themselves with the data by independently and simultaneously reading the transcripts and making notes regarding preliminary codes [37]. The use of multiple coders supported confirmability [36]. Next, each researcher analyzed the entire dataset and collated their preliminary codes into potential themes and subthemes [37]. Following this step, the two researchers then met to discuss and define the themes and subthemes. During this process the researchers ensured that the themes and subthemes were representative of the coded extracts and the entire dataset; this involved refinements to the themes and subthemes and working definitions to create a final codebook [37]. Next, the two researchers re-analyzed the transcripts using the agreed upon codebook. Once qualitative analysis was complete the Qurikos files were merged and exported. To support dependability and potential transferability of the data, the methods, study procedures, and participant characteristics have been documented in detail to enable researchers to replicate the current study and determine if the study findings are relevant for their participants [36].

## Results

### Quantitative

#### Demographics

In the quantitative survey, a total of 374 students participated (*n* = 86 parents, M_age_ = 34.69; *n* = 288 non-parents, M_age_ = 22.41). The majority of non-parent students were pursuing a Bachelor’s degree (*n* = 190; 65.97%), while most of the student-parents were pursuing a Master’s or Doctorate (*n* = 46; 53.48%). Approximately half of the participants were of European origins (*n* = 36; 41.86% parents; *n* = 144, 50% non-parents), and most identified as female (*n* = 57, 66.28% parents; *n* = 195, 67.71%). Most of parent and non-parent students were employed to some extent (*n* = 49, 56.98% parents; *n* = 172, 59.73%) and approximately half of parents reported having an annual household income greater than $75,000 (*n* = 36, 45.35% parents), while approximately half of non-parents reported having an annual household income of $75,000 or less (*n* = 154, 53.48%). Most parents reported being married, common law, or engaged (*n* = 66, 76.74%), while the majority of non-parents reported being single (*n* = 288, 79.17%). Full demographic details can be found in Yates and colleagues [26].

In the focus groups, 10 parents and 10 non-parents participated, with average ages of 35.78 (*SD* = 4.47) and 24.33 (*SD* = 4.03) among parents and non-parents, respectively. The majority of focus group participants were graduate students (90% parents; 60% non-parents), identified as women (60% of parents; 70% of non-parents), and were of European Origins (60% of parents; 40% of non-parents). While most non-parents reported being single (80%) and some reported being unemployed (40%), most parents reported being married, common law or engaged (80%) and the majority were employed (40% employed full-time; 20% employed part-time). Most students attended Western University (100% parents; 70% non-parents) and were enrolled in their studies full-time (70% parents; 100% non-parents). Among parent participants, the average age of their children was 2.83 years (*SD* = 1.47), with most reporting having one child (80%) who lived with them full-time (90%). Full demographic details of focus group participants can be found in Table 1.

**Table 1:** Demographic information–focus group participants.

Participant Characteristics (*N* = 20)	Parents (*n* = 10)		Non-Parents (*n* = 10)	
Age^1^, *M* (*SD*)	35.78 (4.47)		24.33 (4.03)	
	*n*	*%*	*n*	%
**Sex**				
Female	6	60.00	7	70.00
Male	4	40.00	2	20.00
I prefer not to answer	-	-	1	10.00
**Gender**				
Woman	6	60.00	7	70.00
Man	4	40.00	2	20.00
Non-binary	-	-	1	10.00
**Lived Experience as Trans Person**				
Yes	-	-	1	10.00
No	10	100.00	9	90.00
**Ethnicity**				
Asian Origins	4	40.00	3	30.00
European Origins	6	60.00	4	40.00
Multi Racial or Mixed Racial	-	-	1	10.00
Other North American Origins	-	-	2	20.00
**Marital Status**				
Single	-	-	8	80.00
Married, common law, or engaged	8	80.00	2	20.00
Divorced or separated	2	20.00	-	-
**Average Annual Household Income (before taxes)**				
Less than $25,000	2	20.00	1	10.00
$25,000-$50,000	4	40.00	4	40.00
$50,000-$75,000	1	10.00	1	10.00
$75,000-$100,000	1	10.00	-	-
$100,000-$150,000	1	10.00	2	20.00
$150,000-$200,000	-	-	1	10.00
$200,000+	1	10.00	-	-
I prefer not to answer	-	-	1	10.00
**Employment Status**				
Full-time	4	40.00	1	10.00
Part-time	2	20.00	3	30.00
Casual	1	10.00	-	-
Seasonal	-	-	2	20.00
Unemployed	3	30.00	4	40.00
**Post-Secondary Institution**				
Brock University	-	-	1	10.00
Lakehead University	-	-	1	10.00
Niagara College	-	-	1	10.00
Western University	10	100.00	7	70.00
**Degree/Diploma**				
Diploma	-	-	1	10.00
Bachelors	1	10.00	3	30.00
Masters	5	50.00	1	10.00
Doctor of Medicine	-	-	1	10.00
Doctor of Philosophy	4	40.00	4	40.00
**Faculty**				
Arts and Humanities	2	20.00	-	-
Business	1	10.00	-	-
Computer Science	-	-	1	10.00
Education	3	30.00	-	-
Health Sciences	1	10.00	3	30.00
Information and Media Sciences	1	10.00	-	-
Medicine	-	-	1	10.00
Music	-	-	1	10.00
Nursing	-	-	1	10.00
Science	2	20.00	2	20.00
Social Science	-	-	1	10.00
**Enrolment Status**				
Full-time status	7	70.00	10	100.00
Part-time status	3	30.00	-	-

^1^Age measured in years.

#### Mental health

Based on the MHC-SF, the average mental health scores were 20.20 (*SD* = 5.95) and 19.33 (*SD* = 6.23) for parents and non-parents, respectively. Most parents (48.75%) were categorized as flourishing or moderately mentally healthy (43.75%), whereas most non-parents were categorized as moderately mentally healthy (56.72%) according to MHC cut scores. Full details on categorical diagnoses and average scores among parents and non-parents for emotional, social, and psychological wellbeing can be found in Table 2.

**Table 2:** Descriptive statistics of MHC-SF.

Item	Parents’ Mean (SD)	Non-Parents’ Mean (SD)
**Emotional Wellbeing**		
1. How often in the past month did you feel happy?	3.54 (1.04)	3.46 (0.93)
2. How often in the past month did you feel interested in life?	3.88 (1.08)	3.64 (1.19)
3. How often in the past month did you feel satisfied with your life?	3.38 (1.25)	3.14 (1.31)
*Total Emotional Wellbeing Score*	10.79 (2.98)	10.24 (3.05)
*Range of Emotional Wellbeing Scores*	2.00–15.00	1.00–15.00
**Social Wellbeing**		
4. How often during the past month did you feel that you had something important to contribute to society?	3.08 (1.55)	2.83 (1.44)
5. How often during the past month did you feel that you belonged to a community (like a social group, your neighbourhood, your city, your school)?	2.83 (1.62)	3.01 (1.56)
6. How often during the past month did you feel that our society is becoming a better place for people like you?	1.89 (1.55)	2.21 (1.61)
7. How often during the past month did you feel that people are basically good?	2.93 (1.53)	2.74 (1.50)
8. How often during the past month did you feel that the way our society works makes sense to you?	2.03 (1.51)	1.98 (1.62)
*Total Social Wellbeing Score*	12.74 (5.81)	12.76 (5.81)
*Range of Social Wellbeing Scores*	1.00–25.00	1.00–25.00
**Psychological Wellbeing**		
9. How often during the past month did you feel that you liked most parts of your personality?	3.39 (1.29)	3.14 (1.39)
10. How often during the past month did you feel good at managing the responsibilities of your daily life?	3.21 (1.24)	3.09 (1.35)
11. How often during the past month did you feel that you had warm and trusting relationships with others?	3.51 (1.21)	3.44 (1.35)
12. How often during the past month did you feel that you had experiences that challenged you to grow and become a better person?	3.11 (1.50)	3.24 (1.37)
13. How often during the past month did you feel confident to think or express your own ideas and opinions?	3.54 (1.21)	3.35 (1.31)
14. How often during the past month did you feel that your life has a sense of direction or meaning to it?	3.44 (1.43)	3.10 (1.43)
*Total Psychological Wellbeing Score*	20.20 (5.95)	19.33 (6.23)
*Range of Psychological Wellbeing Scores*	3.00–30.00	1.00–30.00
*Total MHC-SF Score*	43.73 (13.41)	42.33 (13.43)
*Range of Total MHC-SF Scores*	7.00–70.00	5.00–70.00
**Categorical Diagnosis**	**Parents’** ***n* (%)**	**Non-Parents’** ***n* (%)**
Flourishing Moderately Mentally Healthy Languishing	39 (48.75%) 35 (43.75%) 6 (7.50%)	100 (37.31%) 152 (56.72%) 16 (5.97%)

Note: Higher scores indicate greater levels of positive wellbeing.

#### Difference between the mental health of parents vs. non-parents

The results from the independent sample *t*-tests indicated no significant differences between the overall scores for mental health, nor emotional, social, or psychological wellbeing of parents compared to non-parents (see Table 3 for full *t*-test results). The results from the tests of proportions revealed no significant differences in the proportion of student-parents classified as flourishing compared to non-parent students (*n*_*parents*_ = 39, *n*_*non-parents*_ = 100, Z = 1.83, *p* = 0.067), and no significant differences in languishing between groups (*n*_*parents*_ = 6, *n*_*non-parents*_ = 16, Z = 0.49, *p* = 0.62).

**Table 3:** Difference between parents and non-parent post-secondary students’ mental health.

Outcome	Group	*n*	M_D_	*df*	*t*-value	*p*	Lower 95% CI	Upper 95% CI
Mental Health	P	80	-1.40	348	-0.82	0.98	-4.76	1.97
	NP	270						
Emotional Wellbeing	P	80	-0.55	348	-1.42	0.90	-1.31	0.21
	NP	270						
Social Wellbeing	P	80	0.02	348	0.03	0.98	-1.43	1.48
	NP	270						
Psychological Wellbeing	P	80	-0.87	348	-1.11	0.49	-2.41	0.67
	NP	270						

Note: M_D_ = mean difference; *df* = degrees of freedom; CI = confidence internal; P = parent group; NP = non-parent group.

### Qualitative

#### Student-Parents

Four themes and four subthemes emerged from the data: (1) factors that challenge mental health (subthemes: worry/anxiety, lack of time); (2) realities of being a student-parent (subthemes: navigating multiple responsibilities, priorities/sacrifices); (3) social connectivity among family and friends; and (4) mental health fluctuations. The definitions of each theme and subtheme can be found in Table 4 and illustrative quotes are in the subsequent paragraphs. Although quotes may be relevant to more than one theme, they are presented in the theme in which the quote best fits.

**Table 4:** Definitions of qualitative themes and subthemes for student-parents.

Theme	Subtheme	Definition
Factors that challenge mental health	Worry/anxiety	Worry and anxieties about family (specifically children), schoolwork, commitments, and/or deadlines.
	Lack of time	A lack of time outside of school and the family unit (e.g., not having enough time to connect with others or prioritize the self).
Realities of being a student-parent	Navigating multiple responsibilities	Navigating being a caregiver, partner, and student as well as the realities associated with separating their role as a parent from their role as student.
	Priorities/sacrifices	Prioritizing being a parent over being a student and the academic sacrifices that are often made as a result.
Social connectivity among family and friends		The importance of social connectivity among family (children and spouse), as well as parents of other children and friends.
Mental health fluctuations		How mental health/wellbeing can change depending on life circumstances (e.g., academic responsibilities, the age of child(ren), environmental stressors).

*Factors that challenge mental health*. Student-parents described factors that challenged their mental health, including worry/anxiety and lack of time. Participants attributed their worry/anxiety to internal pressures to succeed, as well as external pressures (e.g., being a TA, supervisor relationships, taking courses, their role as a parent). While some of the stressors described were related to school, the majority of participants underscored worry/anxiety specific to their child(ren). One participant (N1) emphasized this saying:

I’d say, psychologically, I’m definitely a lot more anxious than I used to be… I mean you’ve got added worries. Yeah, you’re also worrying about your kids, you’ve got people dependent on you and so it’s not just anxiety about school, it’s anxiety about everyone else in your family.

Student-parents emphasized that their concern for their child(ren) outweighed their academic worries/anxiety, as emphasized by another participant (N8) who said, “Anxiety that I tend to experience from non-child related things tend[s] to be very quickly swept aside as soon as there is any child-related emotion or duty that comes up…”. This participant went on to explain that they are able to compartmentalize their worries/anxieties because their priority will always be their child. Additionally, many participants described being worried about their child(ren) getting sick, meeting developmental benchmarks, and socializing with others, which was underscored by one participant (N1) who said:

You worry about them, you know? What they’re doing that day, you want them to have a lot of fun, you want them to be able to get out and meet people and socialize, and then you worry if they’re not making the benchmarks, they’re not talking fast enough or whatever. There’s just a lot more you don’t think about worrying about until you’re a parent.

Another factor that challenged the mental health of student-parents was lack of time. Many participants described having little time for socialization outside of the family unit. Interestingly, this was not viewed as a deficit by student-parents, rather, the majority preferred to spend the little “downtime” that they had with their partner and child(ren). This was underscored by one participant (N10) who said:

I basically have very little time for social interaction and things like that. But at the same time, too, I also find I desire it a lot less… because I’m so much busier, and I’ve got so much going on… When I do have some downtime, I’d rather spend it… with my spouse or doing something like that, rather than going out with friends or doing other sorts of things.

Another participant (N2) echoed this sentiment saying, “I actually prefer not to meet up with people… the PhD Association, they’ll hold events, and I don’t go to any of them. I don’t go to any of those events just because the time needs to be spent elsewhere…” Other student-parents spoke to having little to no time for anything outside of family and academia as emphasized by one individual (N8) who said, “…any time my daughter is sleeping, I’m working on my research or I’m watching lectures, or whatever… there isn’t an opportunity to do anything else.”

*Realities of being a student-parent*. In addition to factors that challenged mental health, participants spoke to the realities of being a student-parent, describing how they navigate multiple responsibilities and competing priorities, as well as the sacrifices they sometimes need to make as a result. Notably, student-parents described the ability to compartmentalize and differentiate their role as a parent from their role as a student. One participant (N10) highlighted this saying:

I think [one] of the advantages of being a student and a parent at the same time is that it allows you to compartmentalize… I think it’s easy to get sucked into grad school, where it becomes this all-consuming beast. But… if I get home later and I come in the door… my daughter comes running up and gives me big hug around my legs… so happy to see me, you know? And [that] just allows me to shift [from] school to… home…

Another participant (N6) shared this sentiment saying, “…you’re able to be pulled out of the negative stuff that happens at… school or work, immediately. There’s the no… time to ruminate on negative experiences, or anything negative at work…” This participant went on to use the analogy of “wearing different hats” and spoke to the challenges of not being able to take the parenting hat off, while another participant (N1) discussed the reality of not letting their academics and mental health impact their child(ren) or parenting.

For many student-parents, they prioritized their role as a parent over their role as student. In doing so, one participant saw an improvement in their mental health, while another participant noted that having a child shifted their perspective as to what was important. This was underscored by one individual (N2) who said, “I’m gonna put my family first, no matter what. If it means getting lower grades, whatever. I mean, I’ll do the best I can do.” Additionally, the majority of student-parents spoke to putting their child(ren) at the forefront, while they prioritized themselves last. This was emphasized by a student-parent (N8) who used a stove top analogy saying:

…there was something that a colleague in my previous career told me about trying to handle all the different responsibilities in your life… you can imagine your life as being… a stovetop with four burners on it, and one burner is for yourself, one burner is for your family, one burner for your career, and one burner is for whatever else there is. The thing about the stove top is that only two burners work at a time. You have to choose which one of those two that are actually working. I think that’s always been a really interesting… a very relevant way of looking at it for me. So since I started this program, my self-burner has been turned off and my career burner and my family burners have been… on full blast the entire time…

Another student-parent (N6) described their tendency to put them self at the end due to “many other things that need to be done.” Some participants reflected on how their priorities shifted as a student before having children to after, as emphasized by one student-parent (N8) who said, “…nowadays I’m much less concerned with being the achiever that I want to be, and I’m more concerned about being the parent that I want to be.” For another individual (N7), being the parent they wanted to be meant putting their mental health first:

…back then [pre-child] it felt literally like life-or-death, like I could not put my work down for anything. So… I think that becoming a parent has improved my ability to put my mental health first, because now I don’t have a choice. If I’m not healthy, if I’m not well, I can’t show up [for] my kid in the way that I want to.

Another participant (N6) described parenting as “the fun part” and everything outside of parenting as a challenge.

*Social connectivity among family and friends*. While many student-parents described having little time for social interaction outside the family unit, participants underscored the benefit of having a social network. Many described their social needs being met through their interactions with other parents, their children and spouse, as well as other student-parents. For example, one individual (N9) spoke to how their social circle primarily consisted of the parents of their children’s friends, while another participant (N2) described their social needs being met by their spouse and child. Some student-parents described their support networks contributing to their child(ren)’s development, as underscored by one participant (N5) who said, “I have different support networks…seeing friends and socializing, that’s hugely helpful in a different way. And I think, based on the development of my child, I rely on different support networks.”

While others underscored the strength in their friendships with parents who are also students saying (N7):

Doing a PhD is uniquely difficult, and being a parent is uniquely difficult, and of the social connections that you might have at this time, many of them might be parents, but they aren’t doing a PhD and they don’t get it, many of them might be doing a PhD, but they’re not parents, they don’t get it. I have a couple of friends who are doing both, they get it in a way that nobody else… So that connection and that solidarity is hugely important…

*Mental health fluctuations*. Student-parents described how their mental health fluctuated depending on life circumstances. Often times this was a result of academic deadlines, their child(ren)’s temperament, and the ability to juggle multiple responsibilities. One individual (N10) underscored the role that their child’s temperament can have on their own mental health saying:

It’s [mental health] up and down per day… like some days… my daughter’s just an absolute delight, and makes a day so much better. Then some days where she’s in the mood and you know it makes a day more difficult because… she doesn’t want to go to day care, she doesn’t wanna brush her teeth, doesn’t wanna go to bed, you know?

Another individual (N10) shared that while some days they feel on top of their tasks and are doing okay, most days they are “struggling to keep their head above water”. Similarly, another student-parent spoke to being forced to function as a parent, despite having competing priorities saying:

…it [mental health] all fluctuates so much, depending on the day, especially in school, because there’s specific deadlines that you’re trying to hit, and you’re balancing things. But I’d say having a child has elevated that even within school, because you’re, it’s a survival thing, you’re forced to function. The household does not function if you’re not functioning, and your child needs everything to function.

#### Non-parent students

Six themes and four subthemes emerged from the data: (1) social connectivity among peers; (2) factors that challenge mental health (subthemes: imposter syndrome, competition, feeling stagnant, lack of social interaction/support); (3) the bidirectional relationship between school and mental health status; (4) prioritizing academic roles; (5) finding purpose through academic pursuits; and (6) admiration for student-parents. The definitions of each theme and subtheme can be found in Table 5 and illustrative quotes are in the subsequent paragraphs.

**Table 5:** Definitions of qualitative themes and subthemes for non-parent students.

Theme	Subtheme	Definition
Social connectivity among peers		The importance of receiving support from various academic communities (virtual vs. in-person).
Factors that challenge mental health	Imposter syndrome	The negative realities associated with comparing oneself to others and relying on academic validation as a measure of success.
	Competition	Academic competition between peers (e.g., grades, job prospects, opportunities).
	Feeling stagnant	The experience of feeling stuck in their degrees, not progressing, and/or experiencing a lack of motivation to continue.
	Lack of social interaction/support	Lack of social interaction/support from peers resulting in feelings of isolation.
The bidirectional relationship between school and mental health status		How school can negatively impact mental health status, just as how mental health status can negatively impact school.
Prioritizing academic roles		Prioritizing school and academic roles above everything, including basic necessities.
Finding purpose through academic pursuits		How school gives meaning and purpose and is tied to students’ identities and accomplishments.
Admiration for student-parents		Acknowledging the “privilege” it is to solely focus on academic pursuits and not have to juggle multiple responsibilities/roles (e.g., parenting).

*Social connectivity among peers*. Similar to parents, non-parent students described benefits of social interaction. Notably, participants underscored that their emotions and wellbeing were dependant on spending time with and receiving support from peers. Such social interactions prevented non-parent students from feeling isolated and provided encouragement during challenging times. This was underscored by one participant (N16) who said:

I don’t know how I could survive university if I didn’t have the support of all the friends and like the relationship that I’ve made this year, just because they help you, they push you to keep going. I just can’t imagine if like, you don’t have that. You can really feel alone and like by yourself…

Additionally, participants described different forms of social connectivity, as another individual (N12) emphasized the positive impact of an online student community saying:

I have… a wide circle of peers as well both friends and acquaintances. Some of them that I know through the virtual community, I’d say have more social supports…by nature of them being connected to that community. I can check in on them and hear about their day… and if they need support, I can give them like an emoji for a hug. Some of my other friends who are students who aren’t part of that [virtual] network, I feel are more isolated because they’re, you know, doing everything remotely, or they just finished, so they don’t have that classroom connection…

*Factors that challenge mental health*. Similar to parents, non-parent students discussed factors that challenged their mental health; however, in contrast to parents, the described challenges were solely academic-related. Further emphasizing the importance of social connectivity, one of the factors that challenged non-parent students’ mental health was a lack of social interaction/support. Participants noted that without a social circle, it was difficult to deal with the demands associated with being a student and that finding this type of community in school can be challenging. This was highlighted by one participant (N13) who said:

I just moved into a new city, so like I don’t see my family [or] my support groups or my social groups or anything like that anymore. So it’s kinda harder to deal with a huge workload and the lack of social life and also the lack of, like, all those traditional support groups that I used to have…

Similarly, another participant (N16) spoke to the challenges of living far away from family and friends saying, “…being away from your friends or family if you live far [can take a big toll on emotional health]. Like some people are from across the country and I would say September is probably a hard month for everyone…” Other factors that challenged non-parent students’ mental health were imposter syndrome, competition, and feeling stagnant in their degree. Non-parent students discussed how they measured their sense of self based on external success and how they stood in relation to other students academically. This was highlighted by one participant (N20) who said:

…there are aspects of school that I think contribute negatively [to my emotional wellbeing] and some of them are just in my own head, they’re not necessarily practical things that happen at school that make me feel poor. But… sometimes it’s my own… perception of my abilities or imposter syndrome.

Another individual (N11) echoed this sentiment saying, “…sadly enough, comparing myself to others and feeling either at par or better would make me feel flourishing, which I hate to say but it’s true.” Participants described how mentalities such as this were detrimental to their overall wellbeing and happiness, and how comparison was an isolating experience. This was emphasized by one non-parent student (N20) who said:

Part of it is also just when you get into grad school, as much as those people in your classes [are] your colleagues and your friends in some ways, they’re also your competitors cause you’re gonna leave academia one day and you’re all hunting for the same jobs… That can be a bit isolating too, just like you’re constantly asking yourself, am I doing as much work as the person next to me? Cause whoever’s doing the most work for these four years gets the job at the end, right?

Other non-parent students who were further along in their degrees described a lack of motivation and purpose, as a result of being in school for an extended amount of time. This was underscored by one participant (N20) who said, “…I’ve been in school forever [laughter], like when does it end? So sometimes that can be a bit draining.” Another participant (N18) shared this sentiment and described feeling as though they were on a “hamster wheel” with no end in sight. Additionally, non-parent students described feeling “stuck” in the same place and a desire to be done with academia. For one participant (N12) this feeling was heavily related to a lack of fulfillment with their degree saying:

I had a lot more purpose in my dissertation earlier in my PhD, but the longer the PhD goes, the less fulfilling it feels. I feel like I kind of peaked in third year in terms of, like, when I was conducting the research, and then it just became, “I just want this done” mode…

*The bidirectional relationship between school and mental health status*. Unlike student-parents, non-parent students described a strong relationship between school and their mental health, and how their identity is closely linked their role as a student. Participants underscored academic stressors that had a negative toll on their mental health. Notably, one student (N16) described the role that academic validation can play on non-parent students’ emotional wellbeing saying:

I think people that rely heavily on academic validation for their sense of like emotional stability, they’ll probably receive like a really great impact [or] a hit to their mental health once they get to university. Especially being in first year, like your marks are a lot different than they were in high school and for some people that’s like very hard for them to accept. So it can take a big toll on [their] emotional health.

On the opposite side of the spectrum, some individuals highlighted how their mental health can impact their role as a student. One non-parent student (N19) said that at one point their mental health was so poor they dropped out of school; however, as a result of this experience they built their resilience and developed productive coping strategies.

*Prioritizing academic roles*. Many non-parent students described how they prioritized school above everything, including basic necessities such as eating, doing laundry, sleeping, and going to the bathroom. One participant (N12) highlighted this saying, “I have to put laundry on my to-do list or eat dinner as a to-do item or I won’t do it… I’ve got stuff to do… I can spend 60 minutes to cook, that’s 60 minutes I could be doing teaching prep…” Participants described having a difficult time setting boundaries between school and the rest of their lives. Notably, one student (N13) talked about prioritizing their academics above their mental health saying:

I put my academic success above everything else in my life, even if it’s not… the best thing to do cause you know, you’re supposed to set aside time for your mental health… But personally, when I’m having a hard time, since my academics [are] the most important thing to me, I tend to neglect everything else in my life. I really withdraw into myself and I focus 100% on my academics, so I don’t… maintain any connections with family, I would stop talking to people, I don’t eat very much, I even forget to go to the bathroom sometimes … I focus 100% on school to motivate me to feel better but then I do poor in school because of the way my mental health is and then I’m neglecting those other things that I should be doing.

*Finding purpose through academic pursuits*. As described, many non-parent students prioritized their academic roles, which might have been the result of school being the primary purpose/goal for participants. Specifically, non-parent students described school as being something to keep them busy and give them purpose. One participant (N13) underscored that “…when I’m not doing something I feel super bored and my mind just goes running and I start diving into that rabbit hole of, you know, psychological things that you shouldn’t be thinking about yourself…” Many participants emphasized that school provided them with a sense of purpose, as emphasized by another participant (N11) who said:

…I have more of a sense of self when things are busy because I feel like I have something to do, I have a purpose, I feel like I belong doing this thing, whatever it is. Whereas if I don’t have anything to do I’m like stuck wondering like, what is my purpose? I don’t feel like I’m basically worthy of anything cause I’m not doing anything right now…

*Admiration for student-parents*. Non-parent students described having admiration for student-parents, as many underscored that they could not imagine how student-parents navigate both roles; one participant (N12) suggested that student-parents have “superhuman powers”. Interestingly, non-parent students described not having children during school as a privilege, as they were able to focus their full attention on academic pursuits, as emphasized by one participant (N16) who said:

It kind of is a privilege to only have to focus on school… like I focus 100% of my attention on school and work whereas, if I had children or a home that I had to pay for I think I would be a lot more stressed and [couldn’t] give school as much as [my] undivided attention…

Another participant suggested that student-parents might have to make sacrifices at their own expense, to provide for their child(ren), while non-parent students do not have to make the same decisions. Interestingly, this participant (N17) also suggested that “…because they have parental responsibilities maybe they’re able to attach their identity to other things and maybe they find happiness through their parenting responsibilities and… the student identity doesn’t define them as much as others.”

## Discussion

The purpose of this mixed-methods, cross-sectional study was to investigate the mental health of parents (with children/youth aged 0–18 years) compared to non-parents pursuing post-secondary education in Ontario, Canada. Interestingly, there were no statistically significant differences in mental health scores between student-parents and non-parent students, and no significant differences in the proportion of student-parents classified as flourishing or languishing compared to non-parent students. With respect to the qualitative data, both groups described factors that challenged their mental health; however, the factors described by non-parent students were academic-specific, while the factors described by student-parents were primarily child-specific. Additionally, non-parent students spoke to the importance of social connection among peers for support, while student-parents described having their social needs met by their family and friends. Lastly, student-parents emphasized the realities of navigating multiple responsibilities and how their mental health can fluctuate based on life circumstances. Non-parent students spoke to how they prioritize their academic roles, how their academic pursuits give them purpose, as well as the deep admiration they have for parents pursuing post-secondary education.

Parents pursuing post-secondary education have been described as “invisible students”; many of which have underscored structural barriers to being a student-parent [12], stigma [11], and difficulties balancing parenthood and academics [22]. Due to the additional parental responsibilities and stressors described [10–12], it was hypothesized that parents pursuing post-secondary education would have poor mental health relative to their non-parent student counterparts. Interestingly, there were no significant differences in mental health scores between student-parents and non-parent students. This finding differs from the literature, as Yoo [38] investigated the perceived stress and general satisfaction of graduate student-parents (*n* = 145) compared to graduate student non-parents (*n* = 400) in the United States and concluded that student-parents had significantly lower levels of stress and higher levels of general life satisfaction compared to non-parent students. While these findings differ from the quantitative results in the current study, they are not necessarily surprising when situated in the context of the qualitative findings, as student-parents described prioritizing family over school, while non-parent students spoke to the all-consuming nature of academia. Specifically, student-parents emphasized being “pulled out” of the negativity associated with school/work through their interactions with their child(ren). Most student-parents spoke to prioritizing their family and in doing so, placing greater value on their parent identity than student identity. One participant overtly stated that becoming a parent benefitted their mental health. On the opposite spectrum, non-parent students described prioritizing their academics over other domains in their life, including necessities such as eating, sleeping, and going to the bathroom. For many non-parent students, their identities were defined by their student status, which they explained negatively impacted their mental health. Based on their qualitative accounts, it is surprising that there were no significant differences in mental health scores between student-parents and non-parent students. It is possible that the difference in group size (parent vs. non-parent) may have skewed results, resulting in non-significance.

Both parent and non-parent students spoke to factors that challenged their mental health, though these differed based on group. Non-parent students described school-specific stressors including imposter syndrome, competition with peers, feeling stagnant in their degrees, and a lack of social interaction/support. These findings are consistent with the literature on post-secondary student stressors. Specifically, Menard and Chittle [39] conducted a review with the purpose of summarizing factors related to imposter syndrome in the post-secondary student population and concluded that imposter syndrome was negatively associated with students’ self-esteem and mental health. Similarly, Posselt and Lipson [40] found that perceived competition was a risk factor for depression and anxiety among undergraduate college students (*n* = 40,350) from 70 institutions. On the opposite spectrum, student-parents underscored worry/anxiety, primarily for their children, and having a lack of time for anything outside of family and school. These findings are consistent with Garbutt and colleagues [41], who explored United States parents’ (*n* = 1,119) health concerns for their children and concluded that parents worried about their child(ren)’s mental health, healthy growth and development, and safety, among other concerns. Additionally, van Rhijn [20] conducted a qualitative study (*n* = 10) in Canada to explore how undergraduate student-parents balanced school and family roles. Similar to findings from the current study, student-parents reported time-related challenges, resulting in having to make sacrifices for their children. While there are no peer-reviewed studies focusing on this topic, it is possible that the dichotomy between non-parent student stressors and student-parent stressors in the current study might be explained, in part, by a shift in perspective that often accompanies being a parent. For example, in a lay article published in Today, the author described how before having children their goals and ambitions were self-oriented, whereas after having children they noted a difference in priorities, as their children took the forefront [42]. It is important to note that more research needs to be conducted to assess the extent to which this explanation is valid. Additionally, findings from the current study can be explained by Erikson’s Stages of Psychosocial Development [43]. Notably, due to their younger age, non-parent students in the current study are more likely to fall within Stage 5 (identity vs. role confusion) or Stage 6 (intimacy vs. isolation) in Erikson’s model, which suggests that non-parent students are still finding their sense of self and crafting their personal identity as well as forming intimate, loving relationships with other people [43]. Thus, the focus for non-parent students is on the self, whereas student-parents are likely to fall within Stage 7 (generativity vs. stagnation), which can be characterized by contributing to society through parenthood [43]. Based on Erikson’s Stages of Psychosocial Development, it is thus unsurprising that the factors that challenged the mental health of non-parent students were individual/academic-specific, while student-parents described other-oriented concerns, specifically for their children.

Interestingly, both parent and non-parent students described the benefit of social connectivity, though the interpretation between groups differed. For student-parents, their social support network was primarily derived from their immediate family, other parents, as well as friends. Non-parent students, however, spoke to their connections primarily with other students, which many emphasized supported their emotional wellbeing and reduced feelings of isolation. For both groups, having a support network specific to their roles (i.e., student and/or parent) was important. These findings are consistent with the literature, as Alsubaie and colleagues [44] examined the differential impact of social support on post-secondary students’ (*n* = 461) wellbeing and concluded that social support from family and friends was significantly negatively correlated with depressive symptoms. Additionally, social support by family and friends predicted quality of life among post-secondary students [44], suggesting that creating opportunities for social connectivity might be one way to protect the mental health of parent and non-parent students alike. Regarding student-parents specifically, Kensinger and Minnick [11] found that student mothers in their study viewed social support to be important to their educational pursuits. Similar to findings from the current study, student mothers reported that family members (e.g., a parent, spouse) provided the most significant social support [11]. Thus, providing parent and non-parent students with opportunities to connect with members of their specific groups might be beneficial in improving their mental health.

### Limitations

This study is not without limitations. Though the current study was powered to detect meaningful differences between groups, the difference in group size (parent vs. non-parent) may have skewed results. In the future, researchers may wish to monitor recruitment and stop recruitment once the desired sample size is reached for each group. Additionally, the study findings were not representative of all post-secondary students in Ontario, Canada. While the intent was to secure representation across various universities and colleges in Ontario, the study sample included students from 3 universities and 1 college. The lack of institutional representation is largely due to the recruitment strategy, as participants were primarily recruited using the host institution’s mass emailer system. This said, the research team attempted to recruit participants via social media platforms; however, they did not receive much interest. This is likely due to a lack of response from groups/organizations when contacted asking to share the recruitment materials with their networks. In the future, researchers may wish to follow-up with social media group administrators, as only one attempt was made per group in the current study. Further, researchers may wish to contact universities/colleges directly and receive ethics approval to recruit using their in-house resources to secure adequate representation of students. Additionally, the majority of participants in the study were pursuing an undergraduate or graduate degree at the host institution, were female-identifying, and many were of high socioeconomic status and European origins. Thus, it is advised that researchers in the future aim to recruit a more diverse sample of students. Additionally, the number of focus groups and focus group participants limits the transferability of study findings [35]. Few participants that completed the survey were also interested in participating a focus group. While data sufficiency was reached, the research team was not able to confirm it (by conducting an additional focus group for each group), as there was a lack of interest from participants. In the future, researchers may wish to recruit a larger and more diverse sample size in an effort to increase the pool and representation of potential focus group participants. Researchers might also consider offering the focus groups at a different time during the year, as it was expressed by focus group participants in the current study that the month of March (during which focus groups were conducted) is notoriously busy for students. Further, the study was at risk for social desirability bias. Specifically, one of the qualitative themes that emerged from the non-parent focus groups was admiration for student-parents. It is possible that this theme was a result of the study purpose and focus group guide. This said, to mitigate social desirability, at the beginning of the surveys and each focus group individuals were reminded that there were no right or wrong answers, that the research team was interested in what was true for them, and they could refuse to answer any questions they wished. Lastly, the focus group participants primarily consisted of students pursuing graduate degrees (for both the parent and non-parent focus groups). Although all of the qualitative themes emanated from focus group transcripts and were de-identified, some themes were especially relevant to the graduate student population. As a result, not all themes were representative of the post-secondary student population in general. In the future, researchers may wish to conduct separate focus groups based on degree type and/or utilize purposive sampling to obtain a more fulsome representation of students.

## Conclusion

The purpose of this paper was to investigate the mental health of parents (with children/youth aged 0–18 years) compared to non-parents pursuing post-secondary education in Ontario, Canada. Findings revealed no significant differences in mental health scores or the proportion of those classified as flourishing/languishing between student-parents and non-parent students. However, student-parents and non-parent students described their stressors in different ways. Specifically, student-parents described placing greater value on their family than academic pursuits, while for many non-parent participants their identity as a student was at the forefront. Both parent and non-parent students described challenges to their mental health, including academic and parental barriers, and spoke to the importance of social connectivity. These findings are particularly noteworthy as, to the authors’ knowledge, the current study is the first to report on the mental health of parents pursuing post-secondary education compared to non-parents. Findings from the current study highlight the dichotomy in student-parent versus non-parent academic identities and are important for university personnel to understand in order to provide tailored supports. Specifically, the study findings might be used to inform institutional policies aimed at reducing structural and systemic barriers for student-parents. This said, it is recommended that researchers build off of the study findings by conducting a larger study with an emphasis on participants from diverse backgrounds who might benefit from a tailored program in a more meaningful way. Notably, student-parents in the current study were primarily female-identifying, married/common law/engaged, and employed with a high annual household income. Thus, researchers may wish to conduct a study focusing on single student-parents of low socioeconomic status or parents who want to pursue higher education but do not have the supports in place to do so, thus aiming to reduce challenges/barriers and make post-secondary education a reality for the majority.

## Supporting information

S1 Appendix Semi-structured focus group guide (parents). (DOCX)

S2 Appendix Semi-structured focus group guide (non-parents). (DOCX)
