# Supplementary material for: The mental health of parent versus non-parent post-secondary students
Source: PLOS Ment Health. 2024 Aug 12;1(3):e0000021. doi: 10.1371/journal.pmen.0000021 (PMC12798575; doi:10.1371/journal.pmen.0000021)
Supplement: S1 Appendix — (DOCX) [file pmen.0000021.s001.docx]

**S1 Appendix**

**Semi-Structured Focus Group Guide (Parents)**

1. Although the specific focus of today’s discussion is mental health, we’d like to start a bit more general. Since we are now into the second semester of the academic year, we’re curious, how everyone is doing in general?

*Earlier in this study we asked a series of questions pertaining to your mental health, inclusive of three wellbeing domains, which we are going to discuss in greater detail. The first wellbeing domain we are going to talk about is emotional wellbeing.*

1. Data analysis revealed that, on a scale ranging from 0-15, parents’ average score for **emotional wellbeing** was 10.79. This means that at the time of data collection parents’ emotional wellbeing was moderately good. (1) To what extent does this feel accurate or inaccurate regarding your own personal experiences of emotional wellbeing? (2) What do you attribute this to (for you and/or for others)?
   1. What would you like to add to this?
   2. Please say more about…
   3. Please elaborate…

*The next wellbeing domain we are going to discuss is social wellbeing.*

1. When we consider participants’ **social wellbeing**, data analysis revealed that, on a scale ranging from 0-25, parents’ average score for **social wellbeing** was 12.74. This means that at the time of data collection parents’ social wellbeing was moderate. (1) To what extent does this feel accurate or inaccurate regarding your own personal experiences of emotional wellbeing? (2) What do you attribute this to (for you and/or for others)?
   1. What would you like to add to this?
   2. Please say more about…
   3. Please elaborate…

*The last wellbeing domain we are going to discuss is psychological wellbeing.*

1. When we consider participants’ **psychological wellbeing**, data analysis revealed that, on a scale ranging from 0-30, parents’ average score was 20.20. This means that at the time of data collection parents’ psychological wellbeing was moderately good. (1) To what extent does this feel accurate or inaccurate regarding your own personal experiences of emotional wellbeing? (2) What do you attribute this to (for you and/or for others)?
   1. What would you like to add to this?
   2. Please say more about…
   3. Please elaborate…

*We are going to shift gears a little bit and discuss our findings regarding participant flourishing and languishing, but before we do this, we want to define the terms for you. Flourishing can be characterized by high positive emotions and high positive functioning. In general terms, this can be understood as positive mental health.*

1. Data analysis revealed that 48.75% of parents were classified as having flourishing mental health. (1) To what extent does this feel accurate or inaccurate regarding your own personal experiences of flourishing? (2) What do you attribute this to (for you and/or for others)?
   1. What would you like to add to this?
   2. Please say more about…
   3. Please elaborate…

*On the opposite spectrum, languishing can be characterized by: low positive emotions and low positive functioning. In general terms, this can be understood as poor mental health.*

1. Data analysis revealed that 7.50% of parents were classified as having languishing mental health. (1) To what extent does this feel accurate or inaccurate regarding your own personal experiences of languishing? (2) What do you attribute this to (for you and/or for others)?
2. What would you like to add to this?
3. Please say more about…
4. Please elaborate…
5. To what extent has your mental health impacted your role as a parent who is also a student, if at all?
6. In what ways has your mental health flourished?
7. In what ways has your mental health languished?
8. To what extent has your role as a parent who is also a student impacted your mental health, if at all?
   1. In positive ways?
   2. In negative ways?
   3. What might be an example of a time where your roles as a student and parent impacted your mental health?
9. What else haven’t I asked you that I should have?

**General Probes:**

- What’s an example of…?
- Please say more about…
- Please elaborate…

**General Definitions** (to be used if asked by participants):

- Emotional wellbeing can be defined as having positive emotion, life satisfaction and interest in life.
- Social wellbeing can be defined as an individuals' appraisals of their experiences in society and includes the five concepts of social contribution, social acceptance, social coherence, social actualization and social integration.
- Psychological wellbeing can be defined as functioning in six domains, including: autonomy, mastery, personal growth, positive relations with others, purpose in life and self-acceptance.
